# SIESTA: enhancing searches for optimal supertrees and species trees

**DOI:** 10.1186/s12864-018-4621-1

**Published:** 2018-05-08

**Authors:** Pranjal Vachaspati, Tandy Warnow

**Affiliations:** Department of Computer Science, University of Illinois at Urbana-Champaign, 201 N. Goodwin Avenue, Urbana, IL, USA

**Keywords:** Phylogenomics, Species trees, Dynamic programming, ASTRAL, NP-hard problems, Supertree methods

## Abstract

**Background:**

Many supertree estimation and multi-locus species tree estimation methods compute trees by combining trees on subsets of the species set based on some NP-hard optimization criterion. A recent approach to computing large trees has been to constrain the search space by defining a set of “allowed bipartitions”, and then use dynamic programming to find provably optimal solutions in polynomial time. Several phylogenomic estimation methods, such as ASTRAL, the MDC algorithm in PhyloNet, FastRFS, and ALE, use this approach.

**Results:**

We present SIESTA, a method that can be combined with these dynamic programming algorithms to return a data structure that compactly represents all the optimal trees in the search space. As a result, SIESTA provides multiple capabilities, including: (1) counting the number of optimal trees, (2) calculating consensus trees, (3) generating a random optimal tree, and (4) annotating branches in a given optimal tree by the proportion of optimal trees it appears in.

**Conclusions:**

SIESTA improves the accuracy of FastRFS and ASTRAL, and is a general technique for enhancing dynamic programming methods for constrained optimization.

**Electronic supplementary material:**

The online version of this article (10.1186/s12864-018-4621-1) contains supplementary material, which is available to authorized users.

## Background

Phylogeny estimation is generally approached as a statistical estimation problem, and finding the best tree for a given dataset is typically based on methods that are computationally very intensive; for example, maximum likelihood phylogeny estimation is NP-hard [1] and Bayesian MCMC methods require a long time to converge. For this reason, among others, the calculation of very large phylogenies is often enabled by divide-and-conquer methods that use “supertree methods” to combine smaller trees into larger trees. A more common use of supertree methods is to combine trees computed by independent research groups on different datasets into a single tree on a large dataset [2]. While Matrix Representation with Parsimony (MRP) [3, 4] is the most well known supertree method, other supertree methods have been shown to have better accuracy than MRP (e.g., Matrix Representation with Likelihood [5], FastRFS [6], and the recently proposed Bad Clade Deletion supertree method [7]). Supertree methods are an area of active research in the computational phylogenetics community, with new methods introduced frequently and used in a variety of contexts [8–10].

Species tree estimation, even for small numbers of species, is also difficult because of multiple processes that create differences in the evolutionary history across the genome; examples of such processes include incomplete lineage sorting (ILS), gene duplication and loss (GDL), and horizontal gene transfer (HGT) [11]. Species tree estimation is therefore performed using multiple loci from throughout the genomes of the different organisms, and is referred to as “phylogenomics”. One of the standard approaches for species tree estimation is to compute gene trees (i.e., trees on different genomic regions) and then combine the trees together into a species tree under statistical models of evolution, such as the multi-species coalescent (which models ILS), that allow for gene tree heterogeneity. Examples of such “summary methods” (i.e., methods that construct species trees by combining gene trees) that are statistically consistent under the multi-species coalescent model include ASTRAL [12–14], GLASS [15], the population tree in BUCKy [16], MP-EST [17], NJst [18], and a modification of NJst called ASTRID [19].

Summary methods share algorithmic features in common with supertree methods in that both construct trees on the set of species by combining trees on subsets of the species set; the difference between the two types of methods is that in the supertree context, the assumption is that the heterogeneity observed between these “source trees” is due only to estimation error, while in the phylogenomic context the assumption is that source trees can differ from each other and from the species tree due to a combination of estimation error and true heterogeneity resulting from ILS, GDL, HGT, or some other causes. Summary methods and supertree methods are often based on attempts to solve NP-hard problems, and typically use heuristics (a combination of hill-climbing and randomization) to search for optimal trees. While these heuristics can be highly effective on small datasets, they are often very slow and there are no guarantees about the solutions they find.

An alternative approach to the use of heuristic searches is constrained exact optimization, whereby the solution space is first constrained using the input source trees, and then an exact solution to the optimization problem is found within that constrained space. This approach can lead to polynomial time methods (where the running time depends on the size of the constraint space as well as on the input) that can have outstanding accuracy. The first use of this approach was presented in [20], which provided a method to find a species tree minimizing the duplication-loss reconciliation cost given a set of estimated gene trees. Since then, many other constrained exact optimization methods have been developed in phylogenomics for different purposes, including computing trees from maximum likelihood quartet trees [21], constructing species tree from sets of gene trees under gene duplication and loss models [22] or under the multi-species coalescent model [12, 13, 23, 24], improving gene trees given a species tree [25], constructing consensus trees [21], constructing supertrees [6], and extracting a tree from a phylogenetic network [21].

Most of these approaches constrain the search space using a set of “allowed bipartitions”, which we define here. Each edge *e* in an unrooted tree *T* on a set *S* of species defines a bipartition *π*_*e*_ of *S* (also called a “split”), obtained by deleting *e* but not its endpoints from *T*; hence, every tree *T* can be defined by its set of bipartitions $C(T) = \left \{\pi _{e}: e \in E(T)\right \}$. The constraints imposed by these algorithms are obtained by specifying a set *X* of allowed bipartitions so that the returned tree *T* must satisfy that $C(T) \subseteq X$. The set *X* is used to define a set of “allowed clades” (comprised of the halves of the bipartitions, plus the full set of species), and dynamic programming is then used on the set of allowed clades to find an optimal solution to the optimization problem. The set *X* has an impact on the empirical performance, but even simple ways of defining *X* can result in very good accuracy and provide guarantees of statistical consistency under statistical models of evolution [6, 13].

The constrained exact optimization approach has multiple advantages over heuristic search techniques. From an empirical perspective, the dynamic programming approach is frequently faster, and if the constraint space is selected well it is often more accurate than alternative approaches that typically use heuristic searches for optimal solutions. From a theoretical perspective, the ability to provably find an optimal solution within the constraint space is often sufficient to prove statistical consistency under a statistical model of evolution (e.g., under the multi-species coalescent model); hence, many of the methods that use constrained exact optimization can be proven statistically consistent, even for very simple ways of defining the constraint set.

These constrained exact optimization methods typically have excellent accuracy in terms of scores for the optimization problems they address (established on both biological and simulated datasets) and topological accuracy of the trees they compute (as established using simulated datasets). A basic limitation of these methods, however, is that they return a single optimal tree, even though there can be multiple optima on some inputs. This limitation reduces the utility of the methods.

We present SIESTA (Summarizing Implicit Exact Species Trees Accurately), an algorithmic tool that can be used to enhance these dynamic programming methods for finding optimal trees. The input to SIESTA is the set $\mathcal {T}$ of source trees, the constraint set *X* of allowed bipartitions, and a scoring function *w* that assigns scores to tripartitions of the taxon set (and which is derived from the optimization function *F* that assigns scores to trees and the set $\mathcal {T}$, as we show later); SIESTA returns a data structure $\mathcal {I}$ that represents the set $\mathcal {T}^{*}$ of trees that optimize the function *F* subject to the constraint that every bipartition in every tree in $\mathcal {T}^{*}$ is in *X*. This data structure $\mathcal {I}$ enables the user to explore the set of optimal trees in various ways. In this study, we use SIESTA to compute consensus trees, to enumerate the set of optimal trees, to count the number of optimal trees, and to report the frequency of each bipartition in the set of optimal trees.

We explore the impact of using SIESTA with two methods that use dynamic programming for constrained exact optimization: the supertree method FastRFS [6] and the ILS-aware species tree estimation method ASTRAL [13]. We show that using SIESTA to compute a strict consensus tree provides improvements in accuracy (in terms of the topology of the estimated tree) compared to a single optimal tree for both ASTRAL and FastRFS when the number of optimal trees is large enough, and is otherwise neutral. Furthermore, using SIESTA with a modification to FastRFS produces more accurate rooted supertrees than Bad Clade Deletion (BCD), the previous best method for rooted supertree construction [7].

Using SIESTA with ASTRAL, a species tree estimation method that addresses incongruence due to ILS, provides additional benefits. For each optimal tree it returns, ASTRAL provides branch support values based on local posterior probabilities, but these values do not take the other optimal trees into account. We show how to correct these support values to take the full set of optimal ASTRAL trees into account, and enable the calculation of a maximum clade credibility (MCC) tree based on these corrected values. Hence, SIESTA provides a valuable tool for both species tree and supertree estimation, providing distinct advantages over the simplistic use of leading methods for these problems. SIESTA, combined with ASTRAL and FastRFS is available at https://github.com/pranjalv123/SIESTA and the datasets analyzed in this paper are available at [26].

## Methods

### The SIESTA algorithm

SIESTA is designed to work with tree estimation methods that seek optimal solutions within a constrained search space using dynamic programming. Recall that in the constrained optimization approach, the input is a set of source trees (estimated gene trees in the case of ASTRAL, generic source trees in the case of FastRFS) as well as a set *X* of allowed bipartitions of the set *S* of species. Given this set *X* of allowed bipartitions, we define a set $\mathcal {C}$ of “allowed clades” by taking the two halves of each bipartition, and we also include the set *S*; thus, $\mathcal {C} = \left \{A: [A|S \setminus A] \in X \right \} \cup \{S\}$.

We also form a set TRIPS of “allowed tripartitions”, as follows. TRIPS contains all ordered 3-tuples (*A*,*B*,*C*) of allowed clades that are pairwise disjoint, that union to *S*, and where $A \cup B$ is also an allowed clade. We require that *A* and *B* be non-empty, but we allow *C* to be empty.

The purpose of creating this set is that it allows us to perform the dynamic programming algorithm to find optimal solutions for some optimization problems. To see this, consider an unrooted binary tree *T* that is a feasible solution to the constrained optimization problem under consideration. Now root the tree *T* arbitrarily and pick some internal node *v* defining clade *c*. Since *T* is a feasible solution to the optimization problem, all the clades in *T*^(*r*)^ (the rooted version of *T*) are allowed clades, and every node *v* defining clade *c* that is not a leaf has two major subclades *A* and *B* defined by its two children. The 3-tuple (*A*,*B*,*C*) where $C= S \setminus (A \cup B)$ is the tripartition associated to node *v* (equivalently, associated to clade *c*). If *v* is the root of *T*, then *C* will be empty. The set of “allowed tripartitions” is defined to ensure that it includes all 3-tuples that could be formed in this way. Finally, by construction, we consider (*A*,*B*,*C*) and (*B*,*A*,*C*) to be equivalent tripartitions. Similarly, given a rooted binary tree *T*^(*r*)^ on leafset *S*, each non-leaf node *v* in *T*^(*r*)^ defines a tripartition (*A*,*B*,*C*) where *A* and *B* are the clades (i.e., leafsets) below the two children of *v*, and $C = S \setminus (A \cup B)$. We refer to the set of tripartitions of a rooted binary tree *T*^(*r*)^ by $\text {trips}\left (T^{(r)}\right)$.

The objective of the constrained optimization problems is to find an unrooted tree *T*^∗^ on leafset *S* that optimizes a function *F*(·) defined on unrooted trees, subject to *T*^∗^ drawing its bipartitions from *X*. Hence, if we root *T*^∗^, we obtain a rooted tree *T*^∗(*r*)^ in which the non-leaf nodes define allowed tripartitions. ASTRAL and FastRFS are each algorithms that find optimal binary trees for some optimization problem, subject to the constraint that the tree draw its bipartitions from a set *X* of allowed bipartitions. These algorithms reframe the problem by seeking a rooted tree that draw its clades (i.e., subsets of leaves defined by internal nodes) from the set $\mathcal {C}$ of allowed clades, and use the dynamic algorithm design that we will now describe.

For both ASTRAL and FastRFS, it is possible to define a function *w* on allowed tripartitions such that for any unrooted binary tree *T* on leafset *S*, letting *T*^*r*^ denote a rooted version of *T* (obtained by rooting *T* on any edge),

1 $$ F(T) = \sum_{t \in \text{trips}(T^{r})} w(t)  $$

where *F*(*T*) is the optimization score for tree *T*.

The existence of a function *w* that is defined on tripartitions and that satisfies Eq. 1 is the key to these dynamic programming algorithms. Given function *w* that is defined on tripartitions, we define a recursive function *f* that is defined on clades that we can then use to find optimal solutions. We show how to define *f* for a maximization problem; defining it for a minimization problem is equivalently easy.

The calculation of *f*(*c*) for a given allowed clade *c* given *w* and *X* uses the following recursion (phrased here in terms of maximization):

$$\begin{array}{*{20}l} {}f(c) =\left\{ \begin{array}{ll} \max \left\{ f(a) + f (b) + w(a,b,x) | (a,b,x) \in \text{TRIPS}, a \cup b = c\right\}, & |c|>1\\ 0, & |c|=1 \end{array}\right. \end{array} $$

By Eq. 1, *f*(*S*)=*F*(*T*^∗^), where *T*^∗^ is the optimal solution to the constrained optimization problem.

Hence, we can solve the optimization problem using dynamic programming. We compute all the *f*(*c*) from the smallest clades to the largest clade *S*. To construct the optimal solution *T*^∗^, when we compute *f*(*c*) for a clade *c*, we record how we obtained this best score (i.e., we record the unordered pair (*a*,*b*) of clades whose union is *c* achieving this optimal score), and we use backtracking to construct the rooted version of *T*^∗^. Then we unroot the rooted tree.

#### The SIESTA data structure

SIESTA modifies these algorithms so they output a data structure that implicitly represents the set of all the optimal trees.

Specifically, when SIESTA computes *f*(*c*), instead of recording a single split of the clade *c* into two subclades that achieves the optimal score for the clade *c*, SIESTA records all such splits of *c*. We describe the high-level idea of SIESTA by describing how a single optimal tree (all of whose clades are drawn from $\mathcal {C}$) can be represented with pointers, and then show how to extend that to represent all optimal trees.

Let *T* be a rooted binary tree, all of whose clades are drawn from $\mathcal {C}$. *T* can be stored as a collection of nodes, where each node contains either two pointers (one to each of its two children, if it is an internal node) or a taxon label (if it is a leaf node). Equivalently, this representation of *T* can be seen as having pointers from each clade *c* (with at least two species) to a pair of disjoint clades *c*_1_ and *c*_2_, whose union is *c*.

We modify this representation to compactly represent a set of rooted binary trees, as follows. Recall that during the dynamic programming algorithm, all optimal ways of splitting a clade *c* into two clades *c*^′^ and *c*^″^=*c*∖*c*^′^ are determined. Each of these ways of splitting *c* into two subclades is stored in a set $\mathcal {I}(c)$, by having each such split represented by a pair of pointers. In other words, instead of having each clade have a pair of pointers to two subclades, each clade has a set $\mathcal {I}[\!c]$ of pairs of pointers to a potentially large number of subclades. Thus, the SIESTA data structure is the array $\mathcal {I}$ indexed by the clades in $\mathcal {C}$, and each element of the array is a set. Note also that $|\mathcal {I}(c)| \leq |X|$, so that the SIESTA data structure uses *O*(|*X*|^2^) space.

The SIESTA data structure also naturally defines a directed acyclic graph whose nodes are labelled by allowed clades *c* (i.e., elements of *X*), and there is an edge from *c* to *c*^′^ if the set $\mathcal {I}(c)$ contains a pair of pointers, with one pointer pointing to *c*^′^. We will say that *c*^′^ is a child of *c* when there is an edge from *c* to *c*^′^. Given such a representation, it is easy to generate any single optimal tree by following a tree from the root of the SIESTA digraph (i.e., starting with the entry $\mathcal {I}[\!S]$) down to the leaves, and at each clade *x* with at least two elements, picking a pair of its children whose clades union to *x*.

The asymptotic running time of this phase is equal to the asymptotic running time of the original DP algorithm, which is $O\left (|X|^{2} \alpha \right)$, where *α* is the time required to calculate *w* for a single tripartition [12]. Storing the entire data structure requires $O\left (|X|^{2}\right)$ space in the extreme case where every tree has the same score, but in many real-world cases will require less.

#### Using SIESTA

We show how we can use SIESTA in various ways, including counting the number of optimal trees, generating greedy, strict, and majority consensus trees, and computing the maximum clade credibility tree.

##### Counting the number of optimal trees.

We traverse the collection of allowed clades from smallest to largest, calculating for each allowed clade *c* the number optsubtrees(*c*) of optimal rooted binary trees that contain exactly the taxa in *c*. Obviously, optsubtrees(*c*)=1 for all clades of size 1. It is also straightforward to check that the number of optimal rooted binary subtrees on larger clades can be computed by examining all the optimal splits of the clade into two parts. Hence,

$$\begin{array}{*{20}l} {}\text{optsubtrees}(c) =\left\{ \begin{array}{lc} \sum_{(x, y) \in \mathcal{I}[c]} \text{optsubtrees}(x) \cdot \text{optsubtrees}(y), & |c|>1\\ 1, & |c|=1 \end{array}\right. \end{array} $$

The number of optimal rooted binary trees is optsubtrees(*S*), where *S* is the entire set of species. For the algorithms we consider (ASTRAL and FastRFS), all rootings of a particular unrooted tree have the same criterion score, and so this quantity should be divided by 2*n*−3, where *n*=|*S*| is the number of species, to get the number of optimal unrooted trees.

##### Calculating consensus trees.

A particular bipartition [ *c*|*S*∖*c*] is present in fraction *A*_*c*_ of the optimal trees, where

2 $$ A_{c} = \frac{\text{optsubtrees}(c) * \text{optsubtrees} (S\setminus c)}{\text{optsubtrees}(S)}  $$

For *α*≥0.5, the *α*-consensus tree is the unique tree that contains exactly those bipartitions that occur in more than fraction *α* of the optimal trees. For smaller values of *α*, we can still construct a consensus tree, but the set of bipartitions that appear with frequency greater than *α* may not form a tree. To construct the *α*-consensus tree, we sort the bipartitions in descending order by *A*_*c*_, restricted only to those bipartitions [ *c*,*S*∖*c*] with *A*_*c*_>*α*, and construct a greedy consensus tree using this ordering. To calculate a greedy consensus tree, we sort all the bipartitions in descending order of *A*_*c*_ and greedily build a tree from them. The majority consensus tree has *α*=0.5, and so is an example of an *α*-consensus tree. The strict consensus tree can also be computed easily, and contains only the bipartitions that It is easy to see that each of these consensus trees can be computed in $O(|X| \log |X|)$ time.

##### Correct local branch support in an ASTRAL tree.

Recall that ASTRAL-II uses a quartet-based local posterior probability (PP) measure [27] to assign support values to edges. However, when there is more than one optimal tree, the branch support in any individual tree is unreliable, since it does not take the other optimal trees into account. However, SIESTA can modify the branch support values by taking the other optimal trees into account. Specifically, for a given bipartition in a tree *T*, we compute its average support across the set of optimal trees (where an optimal tree without the bipartition contributes a support of zero); this is the corrected support for the bipartition.

##### The ASTRAL Maximum Clade Credibility tree.

A natural optimization problem would be to return the tree whose total corrected branch support (as described above), summed over all the edges of the tree, is maximized. Such a tree is called the Maximum Clade Credibility (MCC) tree, but finding such a tree is an NP-hard problem. We developed a greedy heuristic for the MCC tree, as follows. We use SIESTA to compute every optimal ASTRAL tree, and calculate the corrected local branch support values (as described above). We then compute a greedy consensus of the resulting bipartitions, ranked by these corrected support values. We refer to this as the ASTRAL MCC tree.

### Evaluation protocol

We tested SIESTA in two contexts: in conjunction with FastRFS (a supertree method) and in conjunction with ASTRAL (an ILS-aware species tree estimation method). We use both biological and simulated datasets for these experiments, and on each dataset we examined, we used SIESTA to compute the set of optimal solutions, and to compute consensus trees for these sets of optimal trees. Overall, we examined 1020 simulated and 16 biological datasets (5 supertree and 11 phylogenomic).

**Gene tree estimation.** The simulated supertree datasets (both rooted and unrooted) and all the biological datasets we analyzed came with pre-calculated source trees; for the other datasets (i.e., for the simulated phylogenomic datasets) we used RAxML v8.2.4 [28] to estimate gene trees (using options -m GTRGAMMA -p 12345).

**Supertree methods.** We evaluated the impact of SIESTA on the FastRFS v2.0 supertree method, using several variants of FastRFS that vary in how the constraint set of allowed bipartitions is defined:

- FastRFS _*basic*_, which only uses ASTRAL-II to compute the constraint set,
- FastRFS _*enh*_ (i.e., the enhanced version), which adds the bipartitions from the Matrix Representation with Likelihood (MRL) supertree to its constraint set and also from the ASTRID tree (but only when the internode distance matrix that ASTRID computes is complete), and
- FastRFS _*BCD*_, which adds the bipartitions from the BCD supertree, but can only be used with rooted supertree datasets.

Hence, FastRFS uses other supertree methods (i.e., ASTRAL, MRL, ASTRID, and BCD) to compute the constraint set. We ran ASTRID v1.1 and BCD v1.0.1 in default mode. For ASTRAL-II, we ran a custom variant (available at the github site) where we use ASTRAL v4.7.8 to compute the constraint set of allowed bipartitions, and then our own dynamic programming implementation to find optimal solutions to the quartet support optimization problem. This custom version (which we call SIESTA-ASTRAL) produces exactly the same output species tree(s) as ASTRAL v.4.7.8, and allows us to make a comparison between SIESTA used with ASTRAL v4.7.8 to compute consensus trees and a single ASTRAL 4.7.8. tree. For MRL, we used RAxML v8.2.4 [28], with options -m BINGAMMA -p 12345.

The supertree FastRFS _*enh*_ has already been shown to produce more accurate supertrees than ASTRID, ASTRAL, and MRL, on simulated datasets [6]. However, a new supertree method, BCD, has been developed for use with rooted source trees, and has been reported to be more accurate than FastRFS; hence, we explore these FastRFS variants on supertree datasets with rooted source trees, and we compare these variants to BCD. We then explore the impact of SIESTA on the best variant and determine how it compares to BCD.

**ILS-aware species tree methods.** We evaluated the impact of SIESTA on ASTRAL v4.7.8 on the phylogenomic datasets. We also used ASTRID, v1.1 (another ILS-aware method), but only in the context of providing bipartitions for FastRFS. For the biological datasets, we explored the use of the MCC (Maximum Clade Credibility) tree computed using SIESTA.

**Consensus methods.** For each dataset, we use SIESTA to compute the set of optimal trees and then also to compute three consensus trees: the strict consensus, the majority consensus, and the greedy consensus. The strict consensus tree is the unique tree whose bipartition set is exactly those bipartitions that appear in every optimal tree, and so will not be fully resolved whenever the number of optimal trees is two or larger. The majority consensus tree is the unique tree whose bipartition set is exactly those bipartitions that appear in a strict majority of the set of optimal trees; unlike the strict consensus, it may be fully resolved even when there are two or more optimal trees. Finally, the greedy consensus tree is obtained by ordering the bipartitions according to their frequency in the set of optimal trees, and then adding them, one by one, in order of their frequency (from most frequent to least frequent) to a growing tree. By design, the greedy consensus may not be unique, but will always refine (or equal) the majority consensus; similarly, the majority consensus will always refine (or equal) the strict consensus.

#### Datasets

**Simulated supertree datasets.** We use two collections of simulated supertree datasets (one with unrooted source trees and one with rooted source trees), each based on the SMIDgen [29] simulation protocol. The unrooted source trees were originally generated for [29], and have been used to explore the accuracy of several supertrees methods [5, 6]; the rooted source tree datasets were generated for [7], and enable a comparison with the BCD supertree method [7], which requires rooted source trees.

We explore the results on the datasets with 100, 500, and 1000 taxa. Each replicate contains one “scaffold” tree and several clade-based trees. The scaffold tree is based on a random sample of the species, and contains 20%, 50%, 75%, or 100% of the taxa sampled uniformly at random from the leaves of the tree. The clade-based trees are based on a clade and then a birth-death process within the clade (and hence may miss some taxa). The original 100-taxon, 500-taxon, and 1000-taxon datasets had 6, 16, and 26 source trees respectively; the number of source trees was reduced to 6, 11, and 16 for the 500-taxon datasets, and 6, 11, 16, 21, and 26 for the 1000-taxon datasets. Sequences evolved down each scaffold and clade-based source tree under a GTR+Gamma model (selected from a set of empirically estimated parameters) with branch lengths that are deviated from the strict molecular clock. Maximum likelihood trees were estimated on each sequence alignment using RAxML under the GTRGAMMA model (with numeric parameters estimated by RAxML from the data), and used as source trees for the experiment. 25 replicates were analyzed for the 100- and 500-taxon model conditions, and 10 replicates were analyzed for each scaffold factor of the 1000-taxon model condition.

**Simulated phylogenomic datasets.** We obtained multi-locus simulated datasets from [13], and then modified them for this study. These datasets were generated by evolving gene trees within species trees (with speciation close to the leaves of the model tree) under the multi-species coalescent (MSC) model using SimPhy [30], and then evolving sequences down each gene tree under the GTR+Gamma model, with branch lengths deviated from the strict molecular clock, using Indelible [31]. Three levels of ILS were generated by modifying the species tree height.

These datasets were then modified for the purposes of this study. These datasets originally had 200 taxa each, but were randomly reduced to 50 taxa each to reduce the running time. The original datasets had variable length loci between 300 and 1500bp, and were truncated for this experiment to 150bp to produce datasets with properties that are consistent with empirical phylogenomic datasets (which frequently have very low phylogenetic signal). Each replicate was evaluated with 5, 10, and 25 loci. We evaluated model conditions where each gene contained all 50 taxa, as well as model conditions where each gene contained 10, 20, or 30 taxa chosen at random from the taxon set. These datasets with 50 taxa had ILS levels that ranged from moderate to very high; we characterize the ILS using the average normalized bipartition distance (AD) between true gene trees and true species trees. The moderate ILS condition has AD =12%, the high ILS condition has AD =31%, and the very high ILS condition has AD =68%. We also generated incomplete gene trees by randomly deleting a specific number of taxa from each gene (so that all genes are incomplete but have the same number of leaves) and then re-estimated gene trees; this allows us to evaluate species tree estimation when not all genes have all the species (i.e., in the presence of “missing data”) [32]. We estimated gene trees using RAxML [28] under the GTRGAMMA model (with numeric parameters estimated by RAxML), and we analyzed 25 replicates for each model condition (defined by the ILS level, number of loci, and amount of missing data).

**Biological supertree datasets.** We analyzed five (all unrooted) supertree datasets from [29]: Marsupials [33], Placental Mammals [34], Seabirds [35], Temperate herbaceous papilionoid legumes (THPL) [36], and Comprehensive papilionoid legumes (CPL) [37] datasets. See Table 1 for detailed information about these datasets.

**Table 1:** Statistics for biological supertree datasets. We show the number of taxa, source trees, and FastRFS _*enh*_ supertrees for each supertree dataset

Dataset	# Taxa	# Source trees	# FastRFS supertrees
Marsupials [33]	267	158	258048
Placental Mammals [34]	116	726	4
Seabirds [35]	121	7	117760
THPL [36]	558	19	5.9 x 10^34^
CPL [37]	2228	39	7.7 10^92^

**Biological phylogenomic datasets.** We analyzed 11 phylogenomic datasets, described in Table 2. Each of these datasets has multiple genes, and each gene has one unrooted binary maximum likelihood gene tree.

**Table 2:** Statistics of the biological phylogenomic datasets. We show the number of taxa, number of genes, and number of optimal trees for ASTRAL

Dataset (publication)	# Taxa	# Genes	# ASTRAL trees
Ferns [44]	85	25	1
Flatfishes [45]	152	23	1
Gallopheasants [46]	18	1479	1
Hymenoptera [47]	21	24	4
Lichens [48]	31	303	1
Louse [49]	15	1101	1
Mammalian [50]	37	424	1
Sigmontidine Rodents [39]	285	11	72
Skinks [51]	16	429	1
Synchaeta [52]	32	27	2
Testudinella [52]	25	27	7

#### Performance criteria.

For the simulated datasets, we compare the topological accuracy of the trees we compute by comparing them to the model species tree or supertree. We use DendroPy v4.0.3 [38] to compute both the false negative (FN) rate and the false positive (FP) rate with respect to the model tree, where the FN rate is the number of bipartitions in the model tree that are missing from the estimated tree and the FP rate is the number of bipartitions in the estimated tree that are not in the model tree, each divided by *n*−3 (the number of internal edges in an unrooted tree) where *n* is the total number of leaves in the model tree. For each basic tree estimation method (i.e., ASTRAL and FastRFS), we also report Delta-Error, which is the difference between the average error rate (i.e., the average of the FN and FP error rates) computed for the tree estimation method and the average error rate of the strict consensus of the optimal trees found by that method. Hence, when Delta-Error is negative, the strict consensus has overall lower error than a single optimal tree. We also report the *F*1 score, which is the harmonic mean of the precision and recall of the estimated trees. For the biological datasets, since topological accuracy cannot be assessed exactly, we describe differences between the consensus trees we compute using SIESTA and trees computed using other techniques. We also report the number of optimal trees for the optimization problems on all the datasets we examine, and the running time used on the biological datasets.

## Results and discussion

### Overview

Experiment 1 explores the use of SIESTA to compute the number of optimal trees found by FastRFS and ASTRAL, as this indicates the potential for SIESTA to improve accuracy by computing consensus trees. Experiment 2 explores how the choice of consensus tree (strict, majority, or greedy) impacts the average topological accuracy of the resulting tree. The next experiments compare the strict consensus tree to a single optimal tree, with Experiment 3 examining FastRFS variants on simulated supertree datasets and Experiment 4 examining ASTRAL on simulated phylogenomic datasets. Experiment 5 examines the use of SIESTA to calculate branch support with ASTRAL and FastRFS on biological datasets, and Experiment 6 evaluates running time issues.

### Experiment 1: computing the number of optimal trees

We used SIESTA to compute the number of optimal trees found by FastRFS and ASTRAL on both the biological and simulated datasets. We explore the differences between FastRFS variants (which depend on how the constraint set is defined) and also between FastRFS and ASTRAL.

**FastRFS variants.** As shown in Table 1, FastRFS _*enh*_ tends to produce large numbers of optimal trees on the biological supertree datasets, and this number tends to increase with the number of taxa and decreases with the number of source trees. On the simulated supertree datasets, both FastRFS _*enh*_ and FastRFS _*basic*_ typically have a large number of optimal trees (Additional file 1: Tables S1 and S2), but FastRFS _*enh*_ generally had a much larger number of optimal trees than FastRFS _*basic*_. In addition, the number of optimal trees for both variants grows with the number of taxa: FastRFS _*enh*_ typically has tens or hundreds of optimal solutions on datasets with 100 taxa, but there are up to 10^18^ optimal FastRFS _*enh*_ trees on datasets with 1000 taxa. The density of the scaffold factor also impacts the number of optimal trees, with fewer optimal trees with the 100%-scaffold factor than with sparser scaffold factors.

**ASTRAL.** ASTRAL showed distinctly different trends. For example, ASTRAL typically only produced a single optimal tree on the biological phylogenomic datasets, as shown in Table 2. We also examined the number of optimal ASTRAL trees on simulated phylogenomic datasets. As shown in Additional file 1: Table S3, when all the gene trees are complete, nearly all the analyses produced only one optimal ASTRAL tree, and when more than one tree was produced it was typically a very small number (often just two). However, there are many optimal ASTRAL trees on the phylogenomic datasets with incomplete gene trees (see Additional file 1: Table S4). Thus, although ASTRAL usually only finds a single optimal tree, it can (in some cases) return a larger number.

**Comparison of ASTRAL and FastRFS variants on the same datasets.** We then compared the number of optimal trees found by ASTRAL, FastRFS _*basic*_, and FastRFS _*enh*_ on the biological supertree datasets. FastRFS _*enh*_ found the largest number, followed by FastRFS _*basic*_, and then by ASTRAL (Table 3). The comparison between FastRFS _*basic*_ and FastRFS _*enh*_ shows that increasing the size of the constraint space for FastRFS results in an increase in the number of optimal trees, which is as expected.

**Table 3:** Number of optimal trees found by FastRFS _*basic*_, FastRFS _*enh*_, and ASTRAL for biological supertree datasets

Dataset (publication)	FastRFS _*basic*_	FastRFS _*enh*_	ASTRAL
Seabirds [35]	17664	117760	24
Marsupial [33]	24576	258048	96
Placental [34]	64	4	4
THPL [36]	2.7×10^18^	5.9×10^34^	1.1×10^11^
CPL [37]	5.4×10^64^	7.7×10^92^	3.9×10^29^

The comparison between ASTRAL and FastRFS _*basic*_, which have the same constraint set, is more interesting, and suggests that the optimization problem solved by ASTRAL tends to have a smaller set of optimal trees than the optimization problem solved by FastRFS. The reason that FastRFS tends to have more optimal solutions than ASTRAL may be that the number of possible FastRFS scores is substantially smaller than the number of possible ASTRAL scores. Specifically, if *n* is the number of species and *k* is the number of source trees, the FastRFS scores are all integers in the range [ 0,(*n*−3)*k*], while the possible ASTRAL scores are integers in the range $[\!0,k{n \choose 4}]$. Therefore, the frequency of multiple trees with the same optimal score is higher for FastRFS than for ASTRAL. However, ASTRAL has by far a much smaller number of optimal trees, and typically has only one optimal tree under conditions where even FastRFS _*basic*_ has at least 10^6^ optimal trees.

Overall, therefore, FastRFS _*enh*_ typically has many optimal trees on supertree datasets, while ASTRAL typically (but not always) has only one optimal tree when given complete gene trees but can have many optimal trees when given highly incomplete gene trees. This means that if we use SIESTA to compute a consensus tree of the optimal trees, this has a greater probability of impacting FastRFS _*enh*_ than ASTRAL, but can also impact ASTRAL when the input dataset has genes that are missing many taxa.

### Experiment 2: comparing different consensus trees computed using SIESTA

We explored the impact of using different consensus methods (i.e., the strict consensus, majority consensus, and greedy consensus) in conjunction with FastRFS _*enh*_ and FastRFS _*BCD*_. We report the difference in average topological error (i.e., the average of the FN and FP error rates) of these consensus trees compared to a single best tree.

For the unrooted supertree datasets, as seen in Additional file 1: Figure S1, for all numbers of taxa and scaffold factors, the three consensus trees of the best FastRFS _*enh*_ supertrees are nearly identical in accuracy, and typically are more accurate than a single best FastRFS _*enh*_ tree. However, there are some cases where the strict consensus has a very slight advantage over the other consensus methods. Additional file 1: Figure S2 shows FN and FP rates separately for the strict consensus of the optimal FastRFS _*enh*_ trees on the unrooted supertree datasets, and how they are impacted by the number of optimal trees. As expected, the FP rates decrease and the FN rates increase as the number of optimal trees increases; furthermore, as the number of optimal trees increases, the decrease in FP rate is substantially larger than the increase in FN rate. As a result, the average of the FN and FP rates decreases with the number of optimal trees.

We then explored the impact of choice of consensus tree on the simulated rooted supertree datasets (where we used FastRFS _*BCD*_); see Additional file 1: Figure S3. On these data, the strict consensus tree had generally the lowest average topological error rate, followed by the majority consensus, and then by the greedy consensus, but all three consensus trees were typically more accurate than a single best FastRFS _*BCD*_ tree.

### Experiment 3: FastRFS-SIESTA vs. FastRFS on simulated supertree datasets

We compare the strict consensus of the optimal FastRFS supertrees (referred to as FastRFS-SIESTA) to a single FastRFS supertree on the simulated supertree datasets. For the unrooted supertree datasets, we use FastRFS _*enh*_, which was shown to provide better topological accuracy than other supertree methods in [6].

**Fig. 1:**
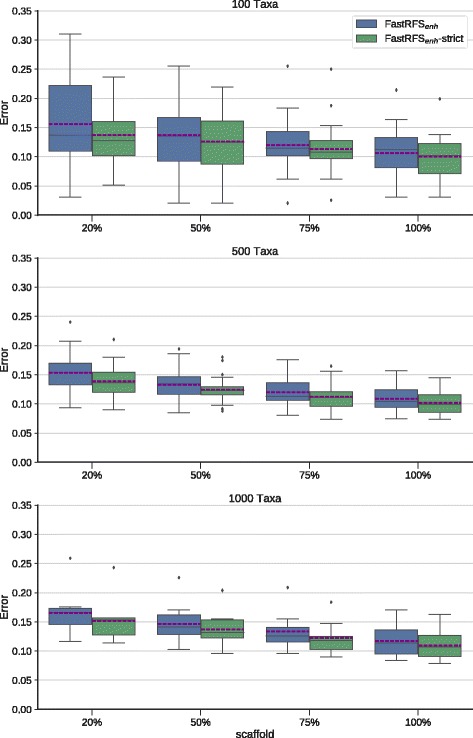
We compare a single FastRFS _*enh*_ supertree to FastRFS _*enh*_+SIESTA (the strict consensus of the optimal FastRFS _*enh*_ supertrees) on unrooted supertree datasets. Error shown is the normalized average topological error (i.e., average of FN and FP rates) between true and estimated supertrees. Error bars indicate the standard error. There are 25 replicates each for the 100- and 500-taxon datasets, and 10 replicates for the 1000-taxon datasets

Results on the unrooted supertree datasets (Fig. 1) show that FastRFS+SIESTA is at least as accurate as FastRFS for all scaffold factors and all numbers of taxa. The difference between the two methods is often small, but there are larger improvements when the scaffold factor is the smallest (which is also when the number of optimal trees is largest).

For rooted supertree datasets, we explore another supertree method called the Bad Clade Deletion (BCD) supertree method, which can only be used with rooted source trees. As shown in [7], BCD produced more accurate species trees (with respect to the F1 metric) than FastRFS _*basic*_ and several other supertree methods. We confirm that BCD outperforms FastRFS _*basic*_ with respect to the F1 metric (Additional file 1: Figure S4), and also note that BCD outperforms FastRFS _*basic*_ with respect to the RF error rate (Additional file 1: Figure S5). However, it is not known whether BCD is more accurate than FastRFS _*enh*_ or FastRFS _*BCD*_, nor whether using SIESTA enables some FastRFS variant to outperform BCD. We compared these three methods with respect to RF errors (Additional file 1: Figure S6) and F1 scores (Additional file 1: Figure S7). The two FastRFS variants are very close in accuracy with respect to both criteria, with a slight advantage to FastRFS _*BCD*_. Interestingly, the comparison to BCD shows that the FastRFS variants are less accurate on the sparse scaffolds than BCD, but slightly more accurate on the 100%-scaffold. Overall, therefore, FastRFS _*BCD*_ has a slight advantage over the other FastRFS variants on these rooted supertree datasets, and is competitive with BCD (worse under some conditions and better under others).

**Fig. 2:**
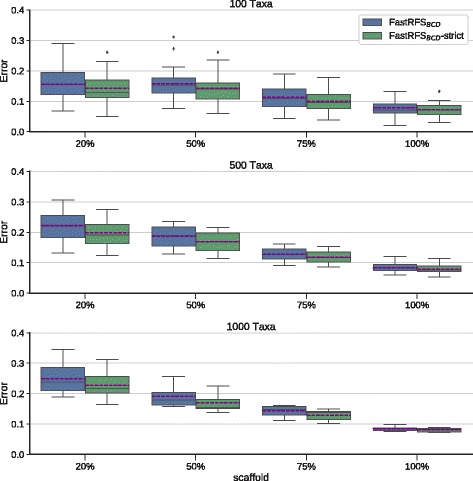
We compare a single FastRFS _*BCD*_ supertree (FastRFS _*BCD*_) to FastRFS _*BCD*_+SIESTA (the strict consensus of the optimal FastRFS _*BCD*_ supertrees) on rooted supertree datasets. Error shown is the normalized average topological error (i.e., average of FN and FP rates) between true and estimated supertrees. Error bars indicate the standard error. There are 25 replicates each for the 100- and 500-taxon datasets, and 10 replicates for the 1000-taxon datasets

**Fig. 3:**
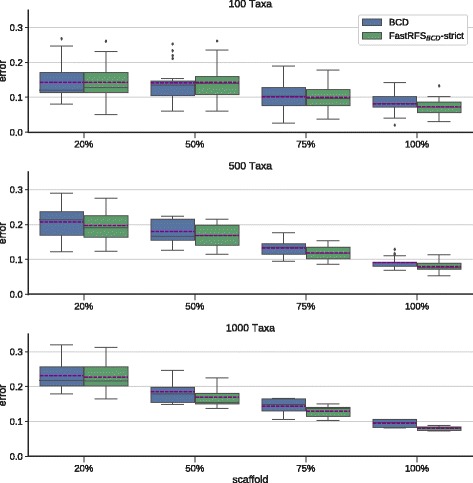
We compare Bad Clade Deletion (BCD) supertrees to the strict consensus of FastRFS _*BCD*_ supertrees on rooted supertree datasets. Error shown is the normalized average topological error (i.e., average of FN and FP rates) between true and estimated supertrees. Error bars indicate the standard error. There are 25 replicates each for the 100- and 500-taxon datasets, and 10 replicates for the 1000-taxon datasets

We then examined whether computing the strict consensus improves FastRFS _*BCD*_ enough to enable it to outperform BCD. We first observed that the strict consensus of the FastRFS _*BCD*_ supertrees was more accurate than a single FastRFS _*BCD*_ supertree (Fig. 2). Furthermore, using SIESTA to compute the strict consensus of the optimal trees found by FastRFS _*BCD*_ produces supertrees that are generally (but not always) more accurate than BCD (Fig. 3 shows average tree error and Additional file 1: Figure S8 shows the F1 scores). The differences are smallest on the 100-taxon datasets, but the strict consensus of the FastRFS _*BCD*_ trees is generally more accurate than BCD on the larger datasets, especially for the denser scaffolds. Thus, the use of SIESTA enables FastRFS _*BCD*_ to outperform BCD.

### Experiment 4: ASTRAL+SIESTA vs. ASTRAL on simulated phylogenomic data

**Fig. 4:**
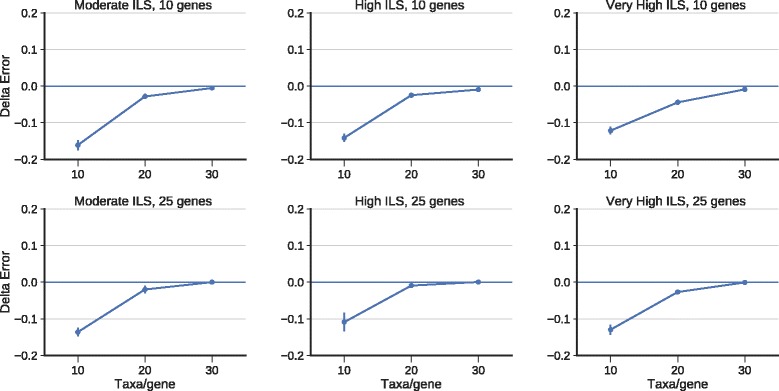
We show Delta-error (change in mean topological error between a single ASTRAL tree and the strict consensus of the set of ASTRAL trees) on simulated phylogenomic datasets with three different ILS levels, 50 species, and 25 incomplete estimated gene trees; values below 0 indicate that the strict consensus of the ASTRAL trees is more accurate than a single ASTRAL tree. We show results for 25 replicates. Error bars indicate the standard error; topological error is the average of the FN and FP error rates

As noted earlier, ASTRAL often returns only one optimal tree, so that the strict consensus of the optimal ASTRAL trees cannot differ from the single best tree. In this experiment, we restrict the attention to the datasets on which ASTRAL found more than one tree. In general, this occurred for the phylogenomic datasets with substantial levels of missing data (i.e., when we deleted species randomly from genes). For these cases, we see that the average topological error rates for the strict consensus of the ASTRAL trees are lower than the error rate for a single ASTRAL tree (Fig. 4) under three different ILS levels, when there is missing data. However, the degree to which the strict consensus of the ASTRAL trees improves over a single ASTRAL depends upon the amount of missing data.

**Fig. 5:**
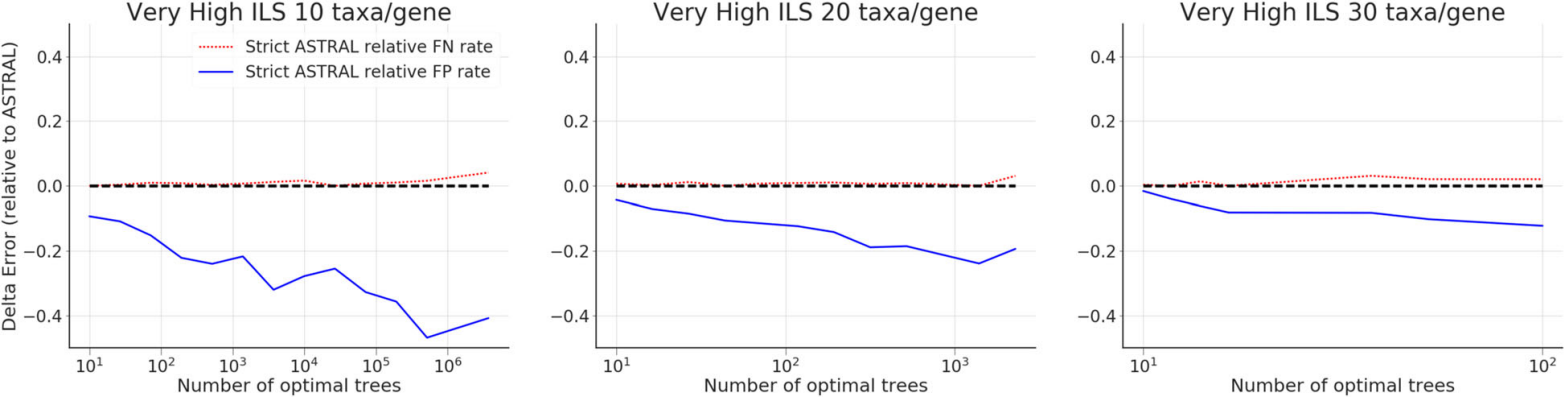
We show the FN and FP error rates of the strict consensus of ASTRAL trees, compared to a single ASTRAL tree, on simulated phylogenomic datasets with 50 species and 25 incomplete estimated gene trees; values below 0 indicate that the strict consensus ASTRAL is more accurate for that criterion (i.e., it has lower error) than ASTRAL. The x-axis shows the number of optimal trees, and we show results for 25 replicates. Error bars indicate the standard error

A more nuanced analysis is shown in Fig. 5, where we explore how the number of optimal trees impacts the FN and FP rates for the strict consensus. Note that the FN rate of the strict consensus is very similar to the FN rate of a single optimal ASTRAL tree, but the strict consensus has a much lower FP rate; hence the strict consensus has a reduced average error rate compared to a single best tree. Although the FN rates are slightly higher under lower ILS conditions, the FP rates drop more than the FN rates increase, so that the same overall trends are similar (Additional file 1: Figure S9).

### Experiment 5: results on biological datasets

For the biological datasets, we do not know the true species tree (which is the unstated objective of the supertree analysis), and so we cannot evaluate accuracy. However, we show how to use SIESTA to provide meaningful branch support in estimated species trees.

**Fig. 6:**
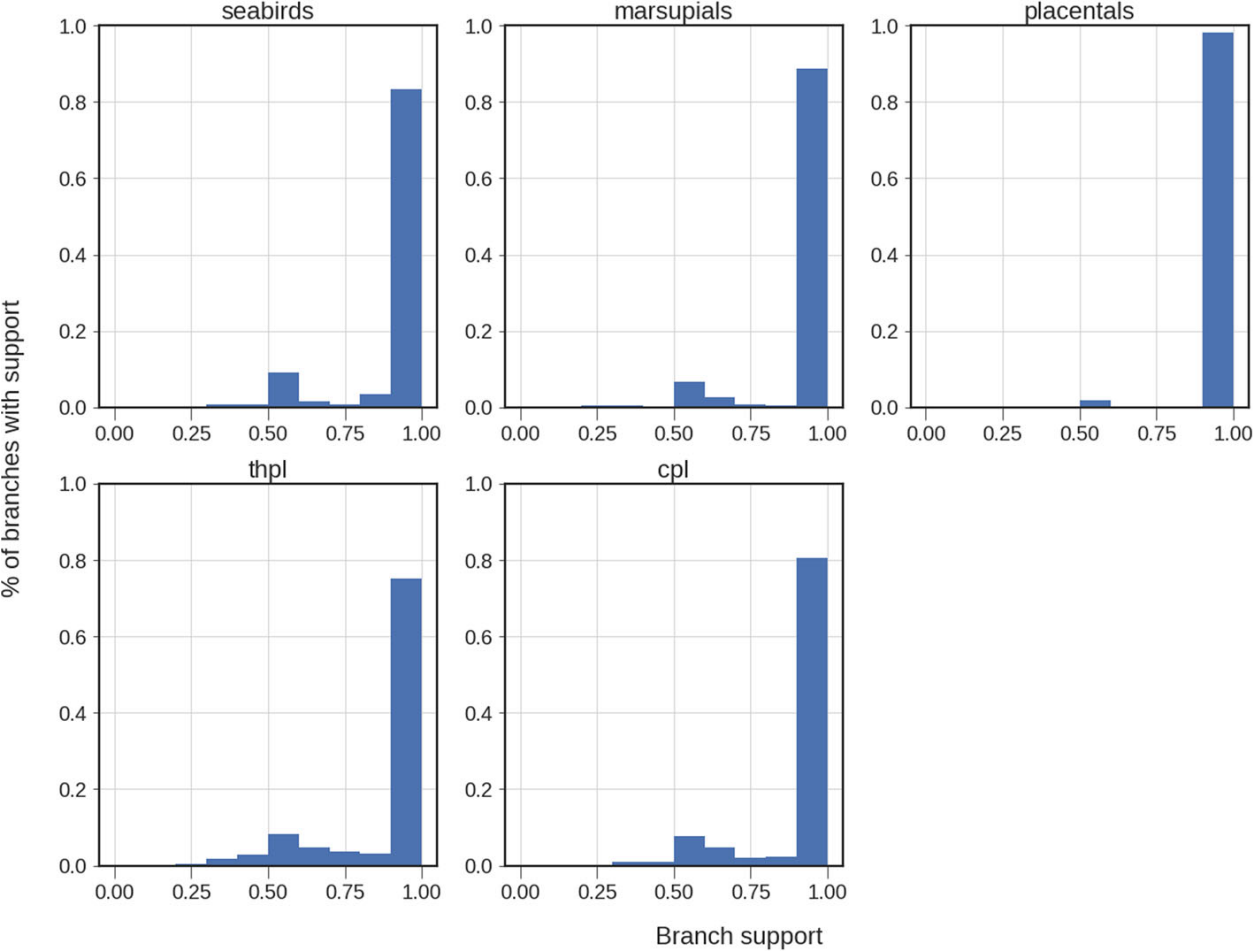
Histogram of support values for edges in the FastRFS _*enh*_ greedy consensus tree on the unrooted supertree datasets. These support values are the percentages of the optimal trees they appear in. Although the majority of the edges have 100% support in each tree, some edges have low support, suggesting that they are not as reliable as the higher support edges

**Biological supertree datasets.** We use SIESTA to compute the greedy consensus tree of the FastRFS _*enh*_ supertrees on the unrooted supertree datasets, and then annotated each edge in the greedy consensus supertree with the fraction of the optimal trees on the dataset. Figure 6 shows that most of the edges in the greedy consensus of the optimal FastRFS _*enh*_ supertrees for each of these datasets have 100% support, indicating that these edges are consistent across all optimal trees. It also shows that some edges are only found in about half (sometimes even less) of the optimal trees, and so should not be considered as reliable. However, this depends on the dataset: nearly all the edges in the greedy consensus of the optimal FastRFS _*enh*_ supertrees for the placental mammals dataset have 100% support, while the THPL and CPL datasets have a substantial fraction of edges that appear in at most 60% of the optimal FastRFS _*enh*_ supertrees.

**Fig. 7:**
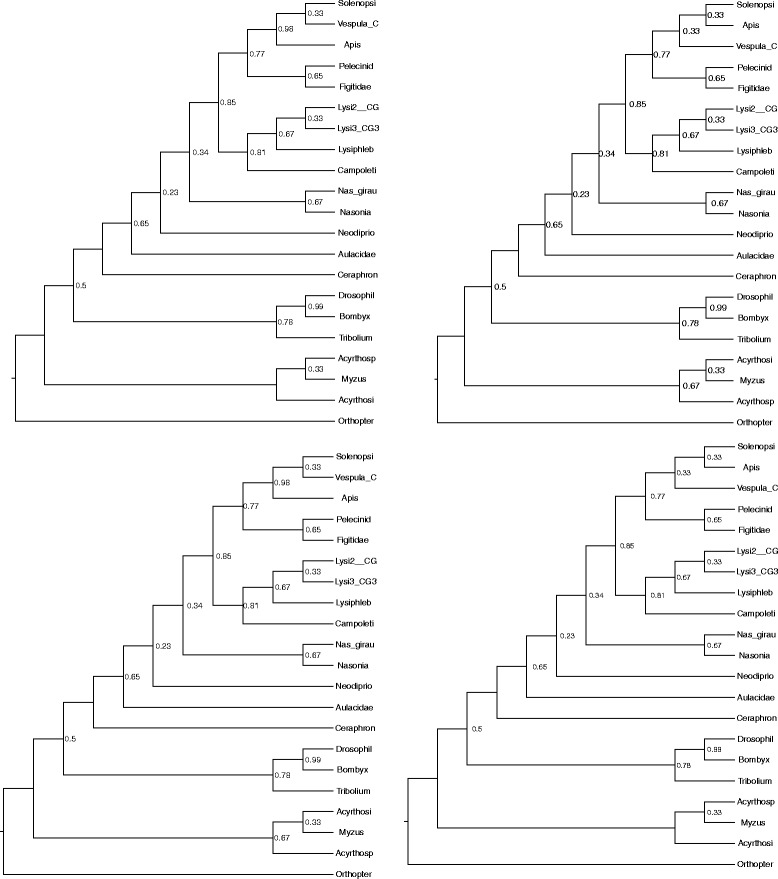
The four optimal ASTRAL trees on the Hymenoptera dataset, each rooted at the outgroup, and given with local posterior probabilities for branch support. The four trees differ only in two groups: (1) Solenopsi, Apis, and Vesputal_C, and (2) Acyrthosi, Myzus, and Acyrthosp

**Fig. 8:**
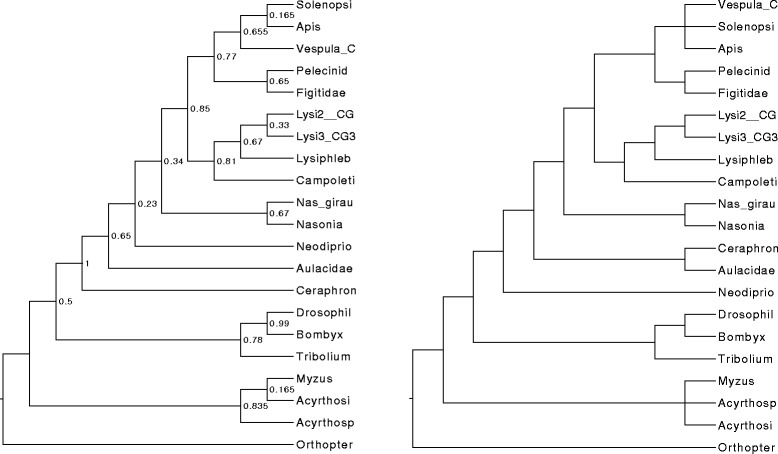
The ASTRAL Maximum Clade Credibility (MCC) tree (left) with branch support and the strict consensus tree (right) on the Hymenoptera dataset. The ASTRAL MCC tree is topologically identical to one of the four ASTRAL trees, but has different branch support; in particular, the branch support on the clades in question is half the branch support in the original ASTRAL trees on these clades. The ASTRAL strict consensus tree makes these two clades into polytomies

**Hymenoptera phylogenomic dataset.** The Hymenoptera dataset is a phylogenomic dataset with 21 taxa and 24 genes. There are four optimal ASTRAL trees on this dataset (shown in Fig. 7). The differences between these four trees are restricted to two clades with three species each: (1) Solenopsi, Apis, and Vesputal_C, and (2) Acyrthosi, Myzus, and Acyrthosp. The strict and majority consensus trees (Fig. 8) on these four ASTRAL trees are identical, and present these two groups as completely unresolved. The MCC tree (Fig. 8) on this set of four ASTRAL trees matches one of the four trees with respect to topology, but has different branch support on the edges, so that the branch support for the two clades in question are halved in comparison to the four ASTRAL trees; thus, the MCC tree appropriately identifies these clades as having very low support.

**Sigmontidine rodent phylogenomic dataset.** The Sigmontidine rodent dataset is a phylogenomics dataset with 285 taxa and 11 genes, and there are 72 optimal ASTRAL trees on this dataset. The species tree computed on this dataset in [39] was a concatenated Bayesian tree using MrBayes [40], with branch support based on posterior probabilities. The Sigmontidine rodent dataset had 72 optimal ASTRAL trees. We computed the ASTRAL MCC tree, and then collapsed all branches with support less than 75%; this produced a tree with only 74 internal edges. This dataset has 285 taxa, meaning that a fully resolved tree would have 282 internal branches. By comparison, the MrBayes tree has 223 internal branches after collapsing branches with less than 75% support.

Comparing the MrBayes tree with the ASTRAL MCC tree, we find that 64 bipartitions are present and highly supported in both trees. After collapsing the edges with lower support, we are left with only the high support edges. Six highly supported bipartitions are present in the ASTRAL MCC tree and compatible with the collapsed MrBayes tree, and three bipartitions are present in the ASTRAL MCC tree and incompatible with the collapsed MrBayes tree. One hundred fifty three highly supported bipartitions are present in the MrBayes tree and compatible with (but not present in) the collapsed ASTRAL MCC tree, and 5 highly supported bipartitions in the MrBayes tree are incompatible with the collapsed ASTRAL MCC tree. The highly supported conflicts between the trees occur in three locations:

1. The MrBayes tree has *Akodon Mimus* as the root of the *Akodon* genus, while the ASTRAL MCC tree has it internal to *Akodon* (the root of *Akodon* is not resolved with greater than 75% support).
2. The MrBayes tree and the ASTRAL MCC tree swap the locations of the *Holochilus* and *Sooretamys* clades, with ASTRAL putting *Holochilus* as the basal clade and MrBayes putting *Sooretamys* as the basal clade.
3. The ASTRAL MCC tree and the MrBayes tree disagree about some resolutions within the *Oligoryzomys* clade.

These placements are in general not well established in the literature [41–43], and so it is not clear which of the two trees is more likely to be correct for these questions.

The difference between a single ASTRAL tree and the ASTRAL MCC tree is therefore quite significant for some datasets. To understand these differences, recall that the support values are obtained using posterior probabilities based on quartet trees around an edge in a single optimal tree. However, a simple example can explain why this can be misleading. Suppose *T*_1_ and *T*_2_ are the only trees that are optimal for ASTRAL, and that *T*_1_ has a split *π* that *T*_2_ does not have. Then under the assumption that *T*_1_ and *T*_2_ are both equally likely to be the true species tree, the *maximum* probability that *π* can be a true split is 0.5 – since it is in only one optimal tree. It is easy to see that any support value greater than 0.5 produced when *T*_1_ is examined is inflated, and that a correction must be made that takes into consideration that *T*_2_ is also an optimal tree. SIESTA’s way of calculating support explicitly enables this correction, since it explicitly considers the support of each bipartition obtained from the entire set of optimal trees.

### Experiment 6: running time

We explore the computational impact of using SIESTA to compute the strict consensus of the optimal trees found using two variants of FastRFS on the rooted supertree datasets with 1000 species. We compare the cost of using FastRFS _*basic*_ to find a single tree to the total running time needed to compute the strict consensus of the FastRFS _*basic*_ supertrees (Table 4). All methods complete in under a minute (actually under 40 seconds), and that the difference in terms of time needed to compute a single FastRFS _*basic*_ tree and the strict consensus of all the optimal FastRFS _*basic*_ trees is at most 0.3 seconds. We also compare the time needed to run BCD, FastRFS _*BCD*_, and the total time needed to compute the strict consensus of the FastRFS _*BCD*_ supertrees (Table 5). Note that BCD is substantially faster than FastRFS _*BCD*_, but that all methods complete in less than a minute. Note also that the difference in terms of time needed to compute a single FastRFS _*BCD*_ tree and the strict consensus of all the optimal FastRFS _*BCD*_ trees is at most 0.5 seconds

**Table 4:** Running time (in seconds, rounded to the nearest tenth) on the 1000-taxon rooted supertree datasets for FastRFS _*basic*_ and for the computation of the strict consensus of the FastRFS _*basic*_ optimal trees (averaged over 10 replicates). The difference in running time to compute the strict consensus of the set of optimal trees compared to computing a single best tree is at most 0.3 seconds

Scaffold factor	FastRFS _*basic*_ (single)	FastRFS _*basic*_ (strict consensus)	Difference
20%	31.6	31.6	<0.1
50%	39.3	39.4	0.1
75%	37.5	37.8	0.3
100%	34.6	34.6	<0.1

**Table 5:** Running time (in seconds) on the 1000-taxon rooted supertree datasets for BCD, FastRFS _*BCD*_, and for the computation of the strict consensus of the FastRFS _*BCD*_ optimal trees (averaged over 10 replicates). The difference in running time to compute the strict consensus of the set of optimal trees compared to computing a single best tree is at most half a second

Scaffold factor	BCD	FastRFS _*BCD*_ (single)	FastRFS _*BCD*_ (strict consensus)	Difference
20%	10.2	33.1	33.5	0.4
50%	8.1	41.8	42.3	0.5
75%	9.2	39.9	40.1	0.2
100%	14.4	36.3	36.4	0.1

Thus, the additional time needed to compute the strict consensus of the set of optimal trees is less than half a second. This is particularly noteworthy, given the number of optimal trees that are found by FastRFS _*basic*_ on these 1000-taxon supertree datasets. Overall, these data show that the cost of using SIESTA is small, and represents a small percentage of the total time needed to find a single tree.

## Conclusions

SIESTA is a simple technique for computing a data structure that implicitly represents a set of optimal trees found during the dynamic programming algorithms used by ASTRAL and FastRFS, but SIESTA is generalizable to any algorithm that uses the same basic dynamic programming structure. Once the data structure is computed, it can be used in multiple ways to explore the solution space. In particular, it can be used to count the number of optimal solutions and determine the support for a particular bipartition, thus enabling the estimation of the support on branches for a given optimal tree that takes into account the existence of other optimal trees.

We studied SIESTA in conjunction with ASTRAL and FastRFS on a collection of biological and simulated datasets. This study showed that using SIESTA to compute the strict consensus produced a benefit for some methods in some cases, but not in all. The trends we observed clearly indicate that when there are many optimal trees, the use of the strict consensus tree results in a substantial reduction in the false positive rate and a lesser increase in the false negative rate, for an overall reduction in topological error. Conversely, when there are only a small number of optimal trees, there is little change between the strict consensus tree and any single optimal tree. Thus, the impact of using the strict consensus depends on the number of optimal solutions, which tended to be larger for all FastRFS variants than for ASTRAL. We also saw that the number of optimal trees for ASTRAL depends on the amount of missing data, so that the benefit of using SIESTA with ASTRAL to compute the strict consensus seems to be reliable only when there is missing data. The study also showed that FastRFS typically benefited from using the strict consensus tree, while ASTRAL’s benefit varied with the dataset, as a result of the differences in numbers of optimal trees.

Our study showed that using SIESTA to produce a maximum clade credibility (MCC) tree with ASTRAL provided a more statistically meaningful point estimate of the true species tree than any single optimal ASTRAL tree, especially with respect to appropriately modified branch support values that take the multiple optima into account. Thus, SIESTA provides multiple benefits to species tree and supertree estimation: identifying cases where there is a unique optimum and providing better point estimates of the true tree when there are multiple optima.

Finally, there are many other methods that also use a dynamic programming approach for tree estimation (often within a constrained search space), and SIESTA can be used with these methods in similar ways. Future work should explore the impact of SIESTA with these other methods.

## Additional file

Additional file 1 Supplementary Materials. Software version numbers and commands. Three tables and nine figures presenting additional results. PDF (935 kb).

## Abbreviations

AD: Average distance
CPL: Comprehensive papilionoid legumes
FN: False negative
FP: False positive
GDL: Gene duplication and loss
HGT: Horizontal gene transfer
ILS: iIncomplete lineage sorting
MCC: Maximum clade credibility
MCMC: Markov Chain Monte Carlo
MRL: Matrix representation with likelihood
THPL: Temperate herbaceous papilionoid legumes
