# Supplementary material for: SIESTA: enhancing searches for optimal supertrees and species trees
Source: BMC Genomics. 2018 May 8;19(Suppl 5):252. doi: 10.1186/s12864-018-4621-1 (PMC5998881; doi:10.1186/s12864-018-4621-1)
Supplement: Supplementary file 1 — Supplementary Materials. Software version numbers and commands. Three tables and nine figures presenting additional results. PDF (935 kb). [file 12864_2018_4621_MOESM1_ESM.xlsx]

SIESTA Supplementary Document

Pranjal Vachaspati Tandy Warnow

January 10, 2018

1 Software Commands and Version Numbers

We provide the detailed commands for the various analyses we performed.

- RAxML v8.2.6 was used to estimate gene trees on the phylogenomic simulated data with arguments "-m GTRGAMMA -p 12345 -n <jobname> -s <input>".
- RAxML v8.2.6 was used to run MRL with arguments "-m BINGAMMA -p 12345 -n <jobname> -s <input>".
- Mrpmatrix (available from `https://github.com/smirarab/mrpmatrix`) was used to calculate the matrix for MRL.
- ASTRAL v4.7.8 was passed to FastRFS and ASTRAL-SIESTA to calculate the search space.
- BCD v1.0.1 was used to calculate BCD trees using arguments "–filetype newick"
- FastRFS v2.0 was used with and without SIESTA, with arguments "–count", "–greedy", "–majority", "–strict", and "–single" used as necessary to count the number of optimal trees, output consensus trees, or output a single optimal tree. The "-e" option was used to pass it additional trees.
- ASTRID v1.1 was used to calculate trees for the constraint set of FastRFS-enhanced, using no additional options.
- Dendropy v4.0.3 was used to calculate error rates with the function

  `dendropy.calculate.treecompare.false_positives_and_negatives`

2 Additional Figures and Tables

taxa	ngenes	scaffold	astral	fastrfs-basic	fastrfs-enh
100	6	20%	9.36	3.52×10^2	1.21×10^3
100	6	50%	4.00	1.31×10^2	1.71×10^3
100	6	75%	1.72	7.27×10^1	1.57×10^2
100	6	100%	1.04	2.49×10^1	3.40×10^1
500	16	20%	1.62×10^3	6.09×10^7	1.96×10^9
500	16	50%	3.94×10^1	1.97×10^8	7.62×10^8
500	16	75%	4.23×10^1	1.37×10^8	6.99×10^8
500	16	100%	1.00	5.36×10^6	2.93×10^7
1000	26	20%	6.48×10^5	2.32×10^{15}	2.50×10^{16}
1000	26	50%	3.60×10^4	9.17×10^{14}	1.11×10^{18}
1000	26	75%	5.67×10^2	2.51×10^{14}	1.68×10^{17}
1000	26	100%	1.00	1.97×10^{13}	5.03×10^{13}

Table 1: Number of FastRFS optimal trees for simulated unrooted supertree datasets. We show the mean number of optimal trees averaged over 25 replicates for 100 and 500 taxa, and 10 replicates for 1000 taxa.

taxa	ngenes	scaffold	fastrfs-basic	fastrfs-bcd	fastrfs-enh
100	6	20%	1.16×10^3	6.06×10^3	4.35×10^3
100	6	50%	5.20×10^2	1.84×10^4	5.19×10^3
100	6	75%	3.00×10^2	1.40×10^3	6.47×10^2
100	6	100%	3.86×10^1	4.95×10^1	4.33×10^1
500	16	20%	4.02×10^{14}	5.42×10^{17}	1.12×10^{16}
500	16	50%	3.57×10^{14}	1.06×10^{22}	9.19×10^{19}
500	16	75%	2.05×10^{12}	7.83×10^{14}	1.65×10^{15}
500	16	100%	4.51×10^7	1.08×10^8	6.55×10^7
1000	26	20%	2.35×10^{29}	4.08×10^{37}	1.28×10^{34}
1000	26	50%	2.80×10^{29}	5.08×10^{36}	2.58×10^{37}
1000	26	75%	2.73×10^{21}	4.27×10^{29}	4.42×10^{27}
1000	26	100%	2.06×10^{14}	4.18×10^{15}	1.54×10^{15}

Table 2: Number of FastRFS optimal trees for simulated rooted supertree datasets. We show the mean number of optimal trees averaged over 25 replicates for 100 and 500 taxa, and 10 replicates for 1000 taxa.

ILS	ngenes	astral
Moderate ILS	5	2.12
Moderate ILS	10	1.12
Moderate ILS	25	1.04
High ILS	5	1.64
High ILS	10	1.00
High ILS	25	1.00
Very High ILS	5	1.20
Very High ILS	10	1.08
Very High ILS	25	1.04

Table 3: Number of ASTRAL optimal trees for simulated 50-taxon phylogenomic datasets. We show the mean number of optimal trees averaged over 25 replicates

ILS	taxa/gene<br>ngenes	10	20	30
Moderate ILS	5	2.87×10^2	7.07×10^2	2.41×10^1
Moderate ILS	10	1.33×10^5	7.01×10^2	1.70×10^1
Moderate ILS	25	1.80×10^7	4.68×10^1	1.80
High ILS	5	1.71×10^2	2.10×10^2	1.55×10^1
High ILS	10	8.17×10^4	6.12×10^2	1.58×10^1
High ILS	25	2.79×10^5	1.03×10^1	1.44
Very High ILS	5	1.76×10^2	1.55×10^2	1.22×10^1
Very High ILS	10	1.67×10^4	1.92×10^2	3.64
Very High ILS	25	1.08×10^5	2.42×10^1	1.40

Table 4: Number of ASTRAL optimal trees for simulated 50-taxon phylogenomic datasets with missing data. We show the mean number of optimal trees averaged over 25 replicates for each model condition.

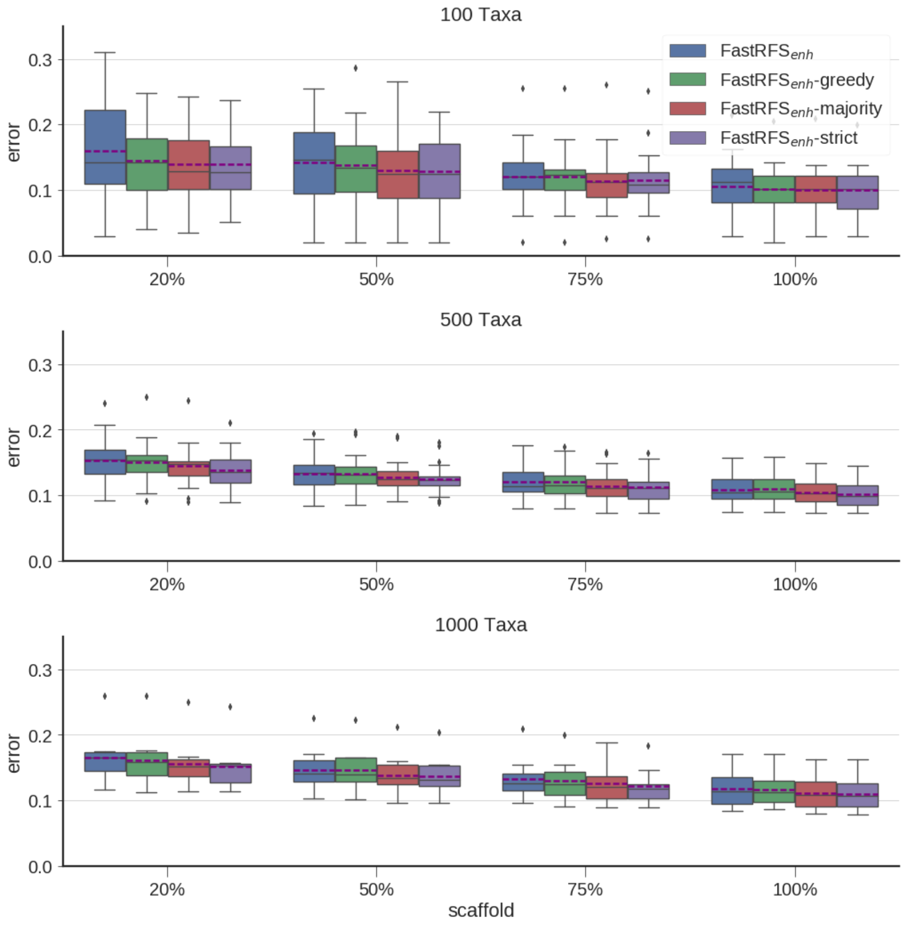

Figure 1: Comparison of average of FN and FP error rates for a single FastRFS$_{enh}$ tree as well as the three consensus trees computed on the optimal FastRFS$_{enh}$ trees on simulated unrooted supertree datasets. We show the mean number of optimal trees averaged over 25 replicates for 100 and 500 taxa, and 10 replicates for 1000 taxa.

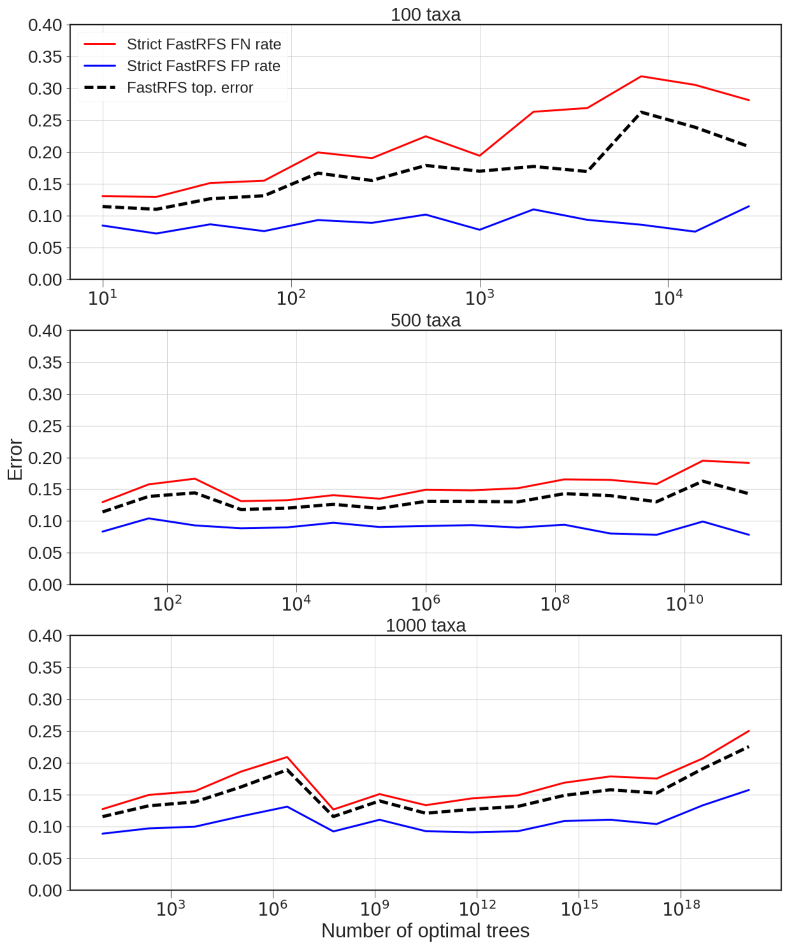

Figure 2: FP and FN rates for FastRFS$_{enh}$ on simulated unrooted supertree datasets as a function of the number of optimal trees. Data gathered from 25 replicates for 100 and 500 taxa, and 10 replicates for 1000 taxa. Red curves show false negative rates; blue curves show false positive rates.

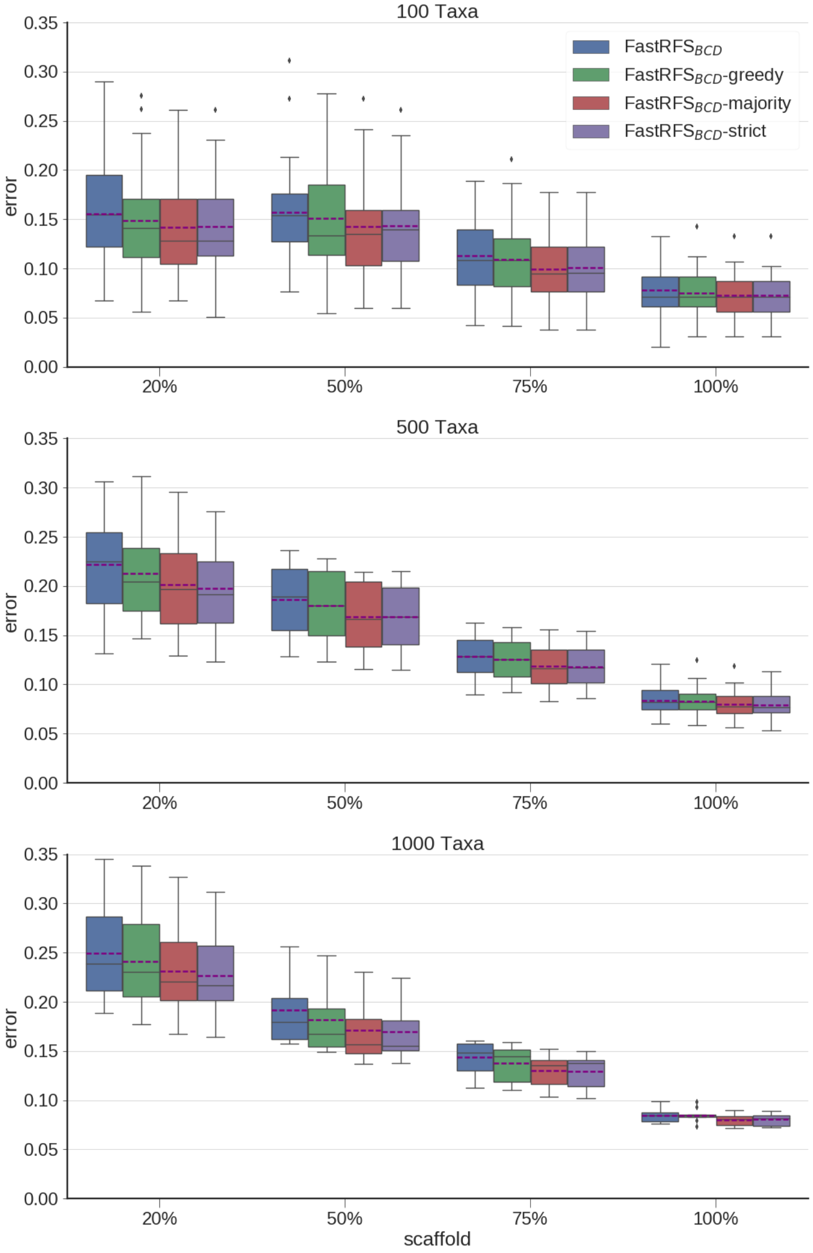

Figure 3: Comparison of average of FN and FP error rates for a single best FastRFS$_{BCD}$ tree and three consensus trees of the best FastRFS$_{BCD}$ trees on simulated rooted supertree datasets. We show the mean error averaged over 25 replicates for 100 and 500 taxa, and 10 replicates for 1000 taxa.

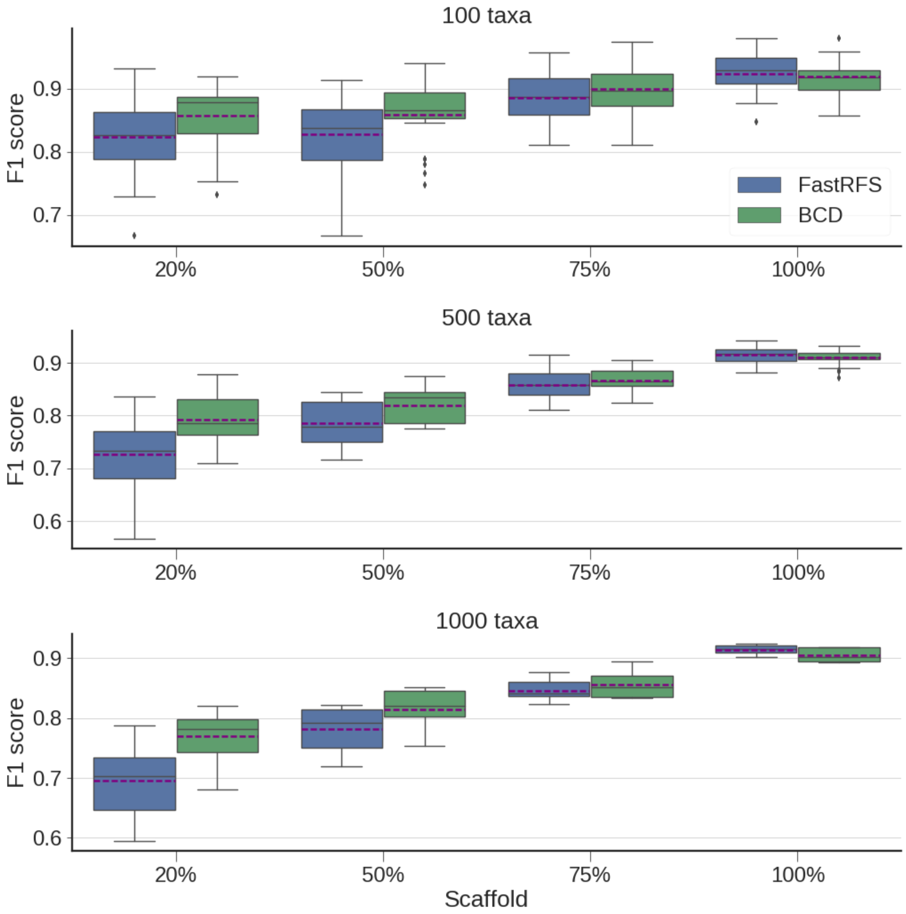

Figure 4: Comparison of F1 scores for a single best FastRFS-basic tree and BCD on simulated rooted supertree datasets. We show the mean scores averaged over 25 replicates for 100 and 500 taxa, and 10 replicates for 1000 taxa.

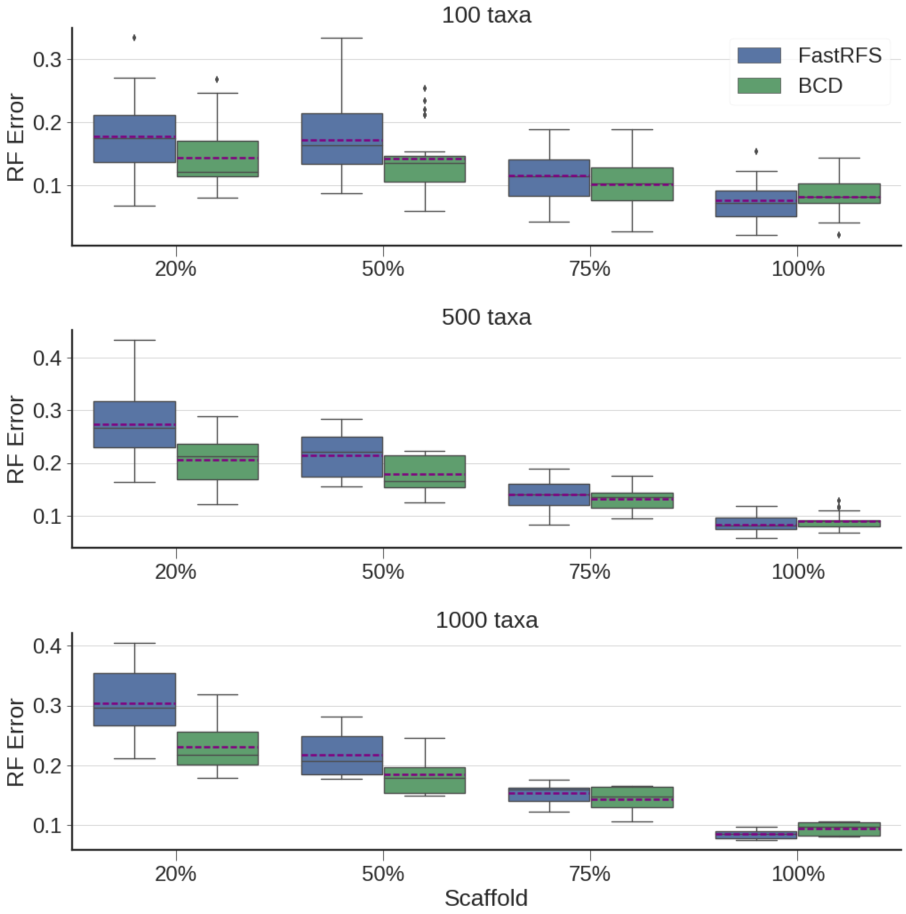

Figure 5: Comparison of average of FN and FP error rates for a single best FastRFS-basic tree and BCD on simulated rooted supertree datasets. We show the mean error averaged over 25 replicates for 100 and 500 taxa, and 10 replicates for 1000 taxa.

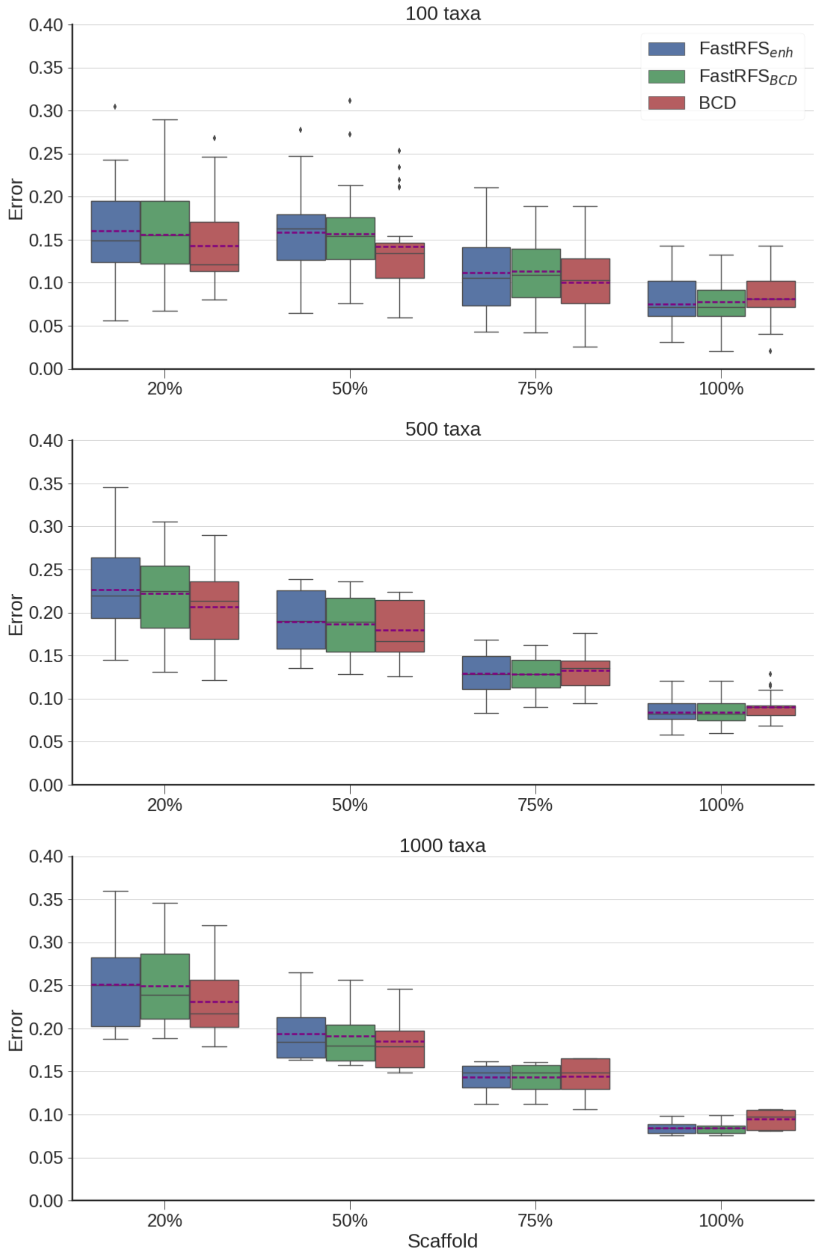

Figure 6: Comparison of average of FN and FP error rates for BCD and the single best trees for FastRFS$_{BCD}$ and FastRFS$_{enh}$ on simulated rooted supertree datasets. We show the mean error averaged over 25 replicates for 100 and 500 taxa, and 10 replicates for 1000 taxa.

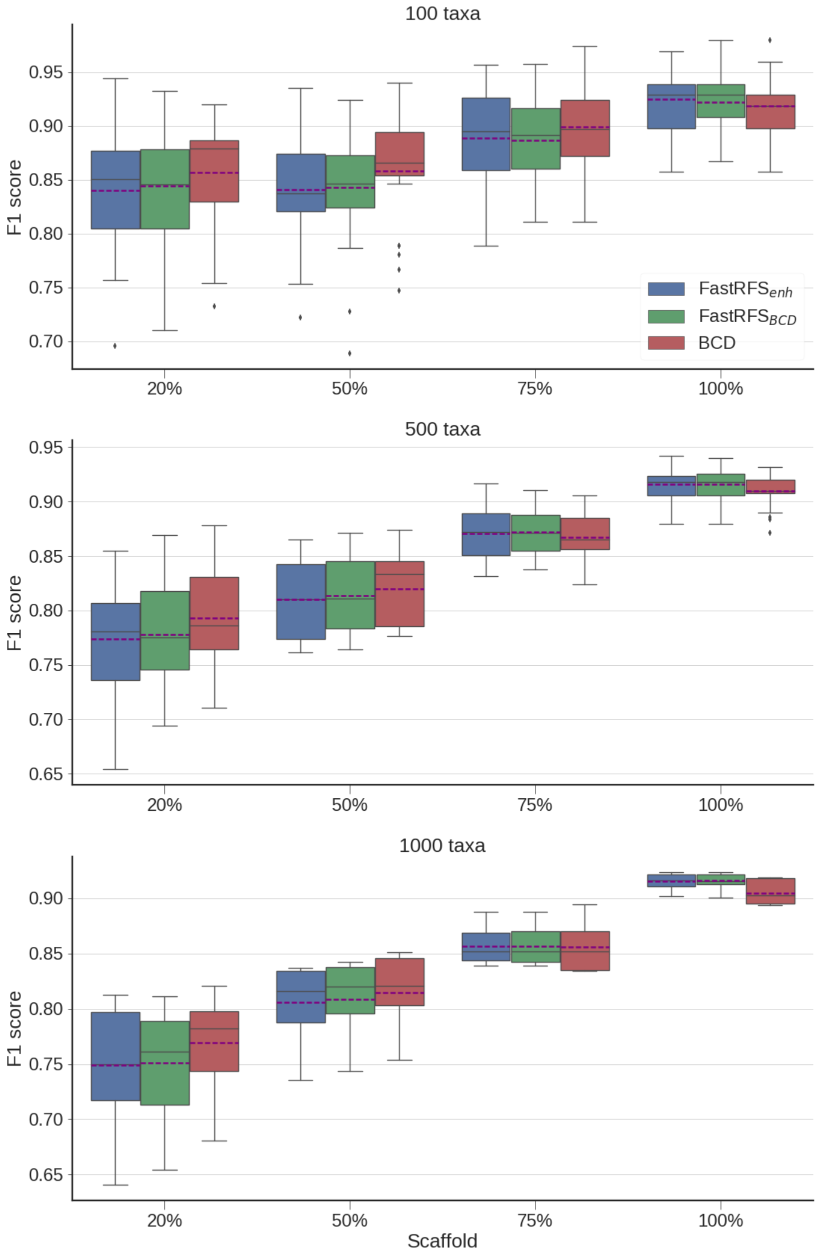

Figure 7: Comparison of F1 scores for BCD and the single best trees for FastRFS$_{BCD}$ and FastRFS$_{enh}$ on simulated rooted supertree datasets.. We show the mean F1 score averaged over 25 replicates for 100 and 500 taxa, and 10 replicates for 1000 taxa.

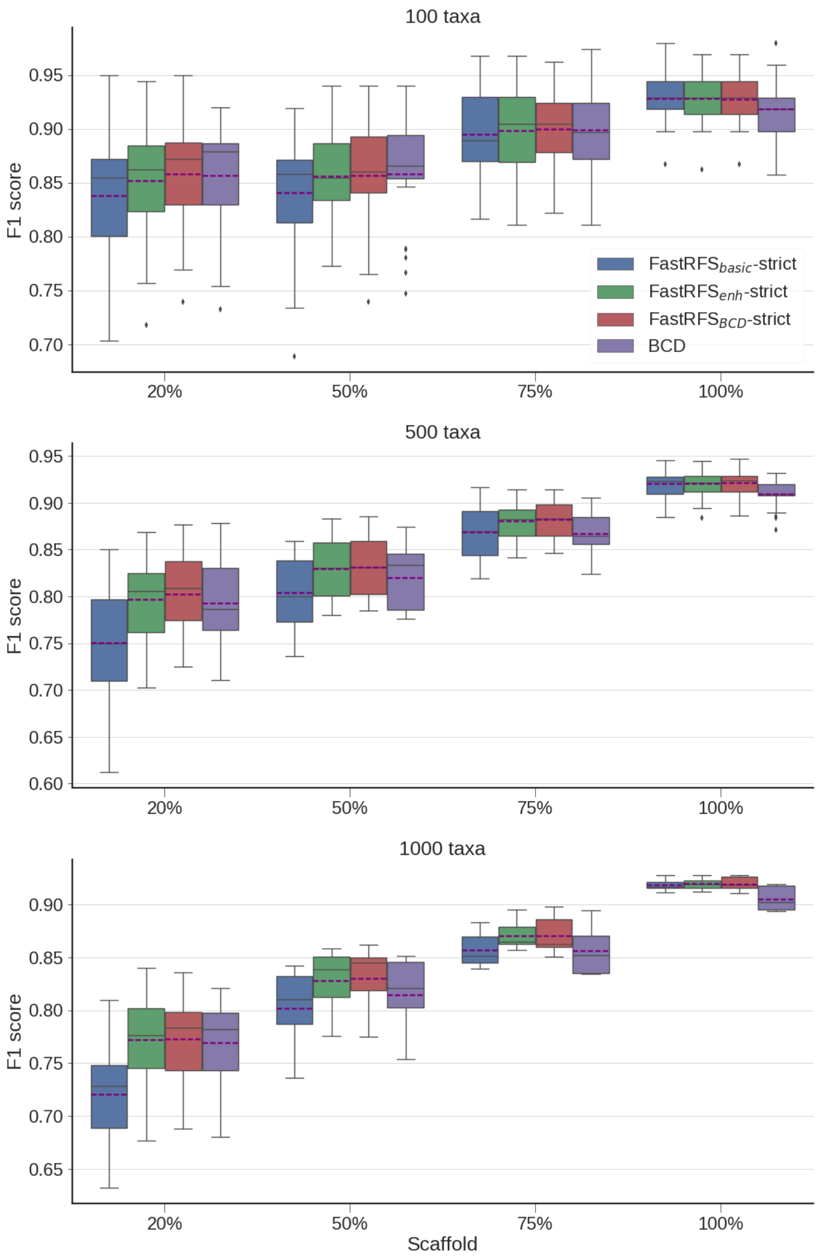

Figure 8: Comparison of F1 scores for the strict consensus of the optimal FastRFS$_{BCD}$ trees and BCD on simulated rooted supertree datasets. We show the mean scores averaged over 25 replicates for 100 and 500 taxa, and 10 replicates for 1000 taxa. Pranjal, restrict to just fastrfs-bcd-strict and bcd.

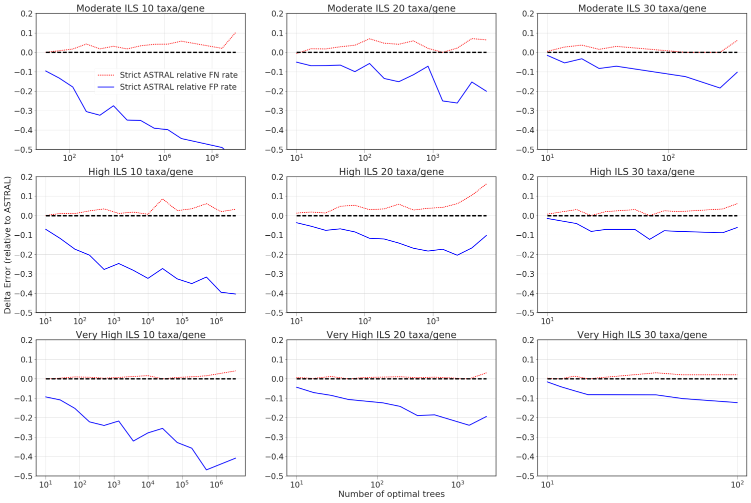

Figure 9: Change in FP and FN rates for the strict consensus of the optimal ASTRAL trees, compared to a single optimal tree, on simulated phylogenomic datasets as a function of the number of optimal trees. Positive values indicate that the strict consensus has a higher error than a single best tree, and negative values indicate that the strict consensus has a lower error than a single best tree. Data are gathered from 25 replicates per model condition. Red curves show false negative rates; blue curves show false positive rates.
